# Pathogen recognition of a novel C-type lectin from *Marsupenaeus japonicus* reveals the divergent sugar-binding specificity of QAP motif

**DOI:** 10.1038/srep45818

**Published:** 2017-04-04

**Authors:** Rod Russel R. Alenton, Keiichiro Koiwai, Kohei Miyaguchi, Hidehiro Kondo, Ikuo Hirono

**Affiliations:** 1 Graduate School of Marine Science and Technology, Tokyo University of Marine Science and Technology, Minato-ku, Tokyo, 108-8477, Japan

## Abstract

C-type lectins (CTLs) are calcium-dependent carbohydrate-binding proteins known to assist the innate immune system as pattern recognition receptors (PRRs). The binding specificity of CTLs lies in the motif of their carbohydrate recognition domain (CRD), the tripeptide motifs EPN and QPD bind to mannose and galactose, respectively. However, variants of these motifs were discovered including a QAP sequence reported in shrimp believed to have the same carbohydrate specificity as QPD. Here, we characterized a novel C-type lectin (MjGCTL) possessing a CRD with a QAP motif. The recombinant MjGCTL has a calcium-dependent agglutinating capability against both Gram-negative and Gram-positive bacteria, and its sugar specificity did not involve either mannose or galactose. In an encapsulation assay, agarose beads coated with rMjGCTL were immediately encapsulated from 0 h followed by melanization at 4 h post-incubation with hemocytes. These results confirm that MjGCTL functions as a classical CTL. The structure of QAP motif and carbohydrate-specificity of rMjGCTL was found to be different to both EPN and QPD, suggesting that QAP is a new motif. Furthermore, MjGCTL acts as a PRR binding to hemocytes to activate their adherent state and initiate encapsulation.

The shrimp immune system relies solely on the primitive innate immune response that includes humoral and cellular responses against infectious agents^1^,^2^,^3^,^4^,^5^. However, evidences of the invertebrate immune system’s capability of discriminating between pathogen at species and even strain level refute the notion that invertebrate innate immune system is naïve in every infection even with the same pathogen^6^,^7^,^8^,^9^. Thus, there is a growing interest in invertebrate immune research in learning how the invertebrate immune system can have high levels of specificity in the absence of antibody-mediated immune responses^6^. Having pathogen-specific immune responses requires a diverse array of pathogen recognition receptors (PRRs) that recognize and initiate the immune response through pathogen-associated molecular patterns (PAMPs)^10^,^11^. Upon recognition of PAMPs, PRRs trigger a cascade of immune responses such as agglutination, encapsulation, nodulation, phagocytosis, the release of antimicrobial peptides (AMPs), and activation of the pro-phenoloxidase (ProPO) system leading to melanization, all of which promote the degradation and clearance of pathogens^12^,^13^,^14^,^15^,^16^,^17^,^18^,^19^. The shrimp immune system has 11 PRRs^20^ and among these are the galectins, *β*-1,3-glycan-binding proteins (BGBPs), *β*-1,3-glycanase-related proteins (BGRPs), and C-type lectins (CTLs), all of which specifically bind to cell surface carbohydrates of pathogens^11^,^20^.

Indeed it is intriguing how carbohydrate complexes from glycans, which holds immensely complex biological information not encoded in the genome, are recognized by PRRs^21^,^22^. It is then fitting to look into the lectins, which bind and recognize carbohydrates. Among animal lectins, CTLs are the most abundant and diverse superfamily, they are lectins containing one or more C-type carbohydrate recognition domains (C-type CRDs) that function in a calcium-dependent manner^21^,^22^. CRDs have been characterized into two groups according to their motif which can be mannose(Man)-binding EPN motif or galactose(Gal)-binding QPD motif 20,23,24. However, variations of these motifs resulting from a mutation of single amino acid were reported, in shrimp CTLs variants include EPK, EPD, EPQ, EPS, QPN, and QPT^20^.

As literature on both CTLs increased, ambiguity and confusion over the use of terminologies for their classification arose^24^. To avoid confusion we conform to the previous review on CTLD superfamily^24^ (24) we will use CTLD-containing proteins (CTLDcps) to refer to proteins containing the carbohydrate-recognition (CRD) domain, which encompasses both the classical Ca^2+^-dependent carbohydrate-binding C-type lectins (CTLs) containing C-type CRDs, and the non-lectin C-type lectin-*like* domains (CTLD).

Recently, another binding motif variant with a QAP (Gln-Ala-Pro) sequence has been reported in several shrimp CTLs. These inlcude CTLDs from *Litopenaeus vannamei* (LvCTLD)^25^ and *Macrobrachium niponense* (MnCTLDcp1,2)^26^ and LdlrLec1,2 from *Marsupenaeus japonicus*^27^. The binding specificity of the QAP motif in LvCTLD, was originally reported to be Gal, suggesting QAP motif has the same specificity as QPD motif 25.

In this study, however, we report a different binding specificity in the QAP motif of a novel CTL from *M. japonicus* (MjGCTL). The present findings provide insights on how CTLs acts as PRRs working as non-self recognition, initiating immune response such as encapsulation by hemocytes.

## Results

### Molecular characteristics of MjGCTL

**Figure 1:**
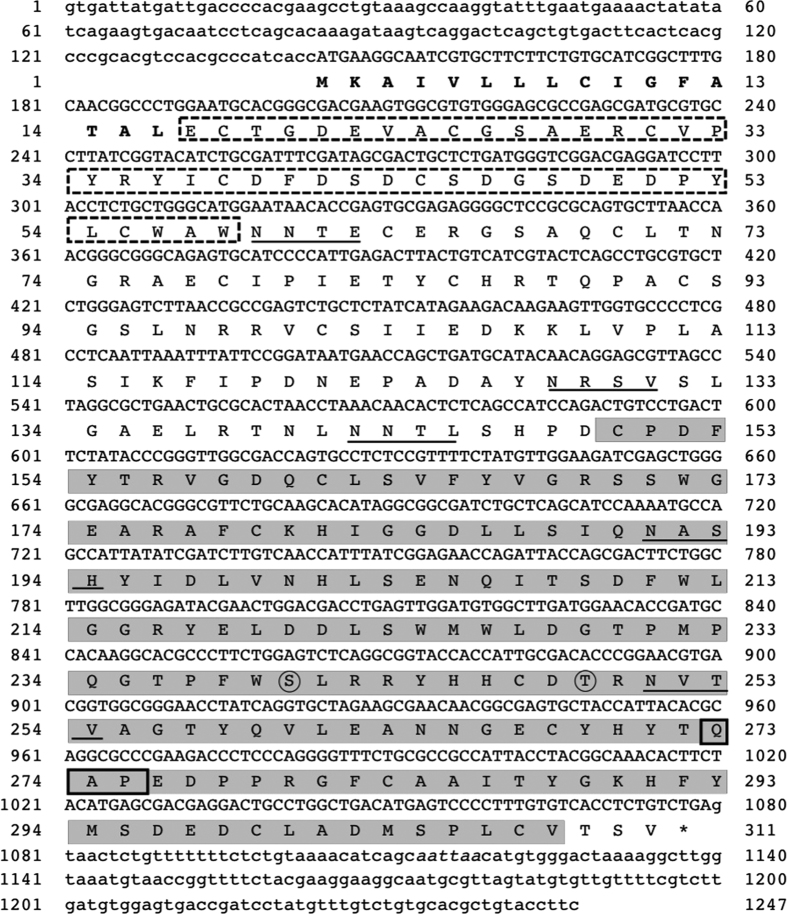
Sequence analysis of MjGCTL. Complete nucleotide cDNA and deduced amino acid sequences of MjGCTL. The signal peptide is bold-faced, and Low-density lipoprotein receptor class A (LDLa) domain is enclosed in a dotted box, the carbohydrate recognition domain is highlighted with its QAP binding motif designated by a solid box. Predicted N- and O- linked glycosylation sites are underlined and encircled, respectively.

**Figure 2:**
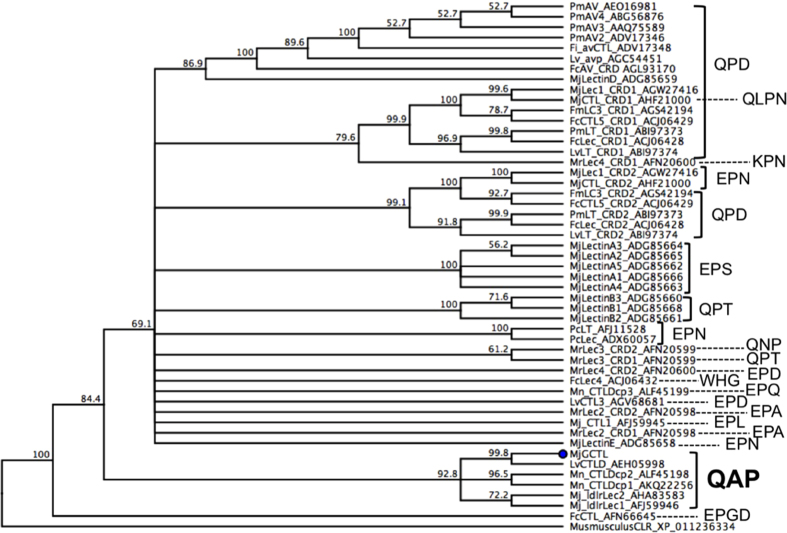
Phylogenetic analysis of shrimp carbohydrate recognition domains (CRDs) with 1000 bootstrap replicates. MjGCTL is represented with a (•). Each CTLDcps are labeled with their respective sugar-binding motifs.

The full cDNA sequence of MjGCTL (DDBJ: LC127418) was obtained. Its ORF was comprised of 935 nucleotide bases in length encoding 311 amino acid residues (Fig. 1), with a predicted molecular weight of 35 kDa. Analysis of protein domains by SMART revealed MjGCTL is comprised of a signal peptide, low-density lipoprotein class-A receptor (LDLa) domain, and a carbohydrate recognition domain (CRD). The predicted amino acid sequence of MjGCTL’s CRD has a QAP binding motif. The MjGCTL amino acid sequence has 5 predicted N-glycosilation and 2 GalNAc O-glycosylation sites (Fig. 1). In the phylogenetic analysis of the amino acid sequences of shrimp CRDs (Fig. 2), MjGCTL clustered with other shrimp CTLs with QAP motifs with a 99.9% bootstrap value.

### Tissue distribution and gene expression profile of MjGCTL

**Figure 3:**
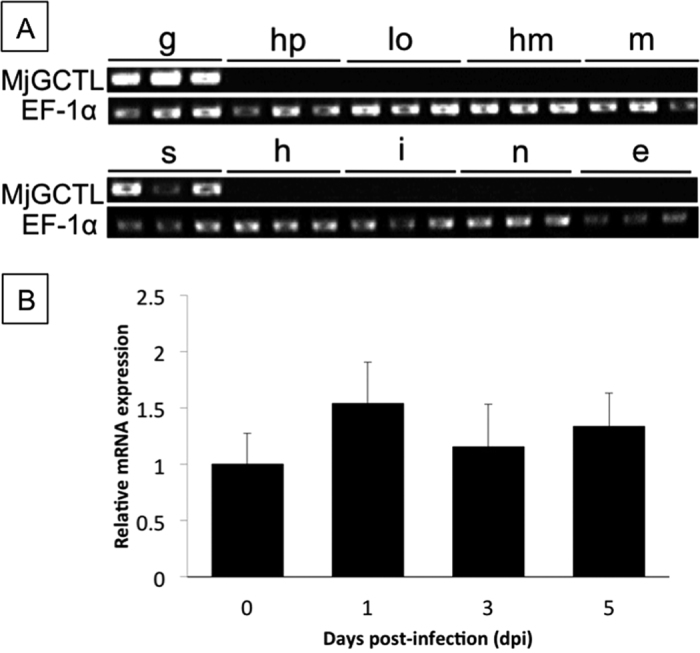
Tissue expression profile of MjGCTL. (**A**) Tissue distribution of MjGCTL by RT-PCR analysis. Transcripts of MjGCTL together with elongation factor 1*α* (EF-1*α*) as internal control was analyzed in gills (g), hepatopancreas (hp), lymphoid organ (lo), hemocyte (hm), muscle (m), stomach (s), heart (h), intestine (i), nerve (n), and eye (e). (**B**) qRT-PCR analysis of MjGCTL mRNA expression levels after 0, 1, 3, 5 days post-infection (dpi) with WSSV. There was no significant difference between sampling time at pvalue 0.05.

MjGCTL transcripts were expressed only in gill and stomach tissues of *M. japonicus*, with expression being strongest in the gills (Fig. 3A) thus we designated the name *M. japonicus* gill C-type lectin (MjGCTL). MjGCTL mRNA relative expression after WSSV infection, analyzed by qRT-PCR, showed that compared to 0 dpi, there were no significant differences among the mRNA levels of MjGCTL at 1, 3, and 5 dpi (Fig. 3B).

### Western Blot analysis of rMjGCTL

**Figure 4:**
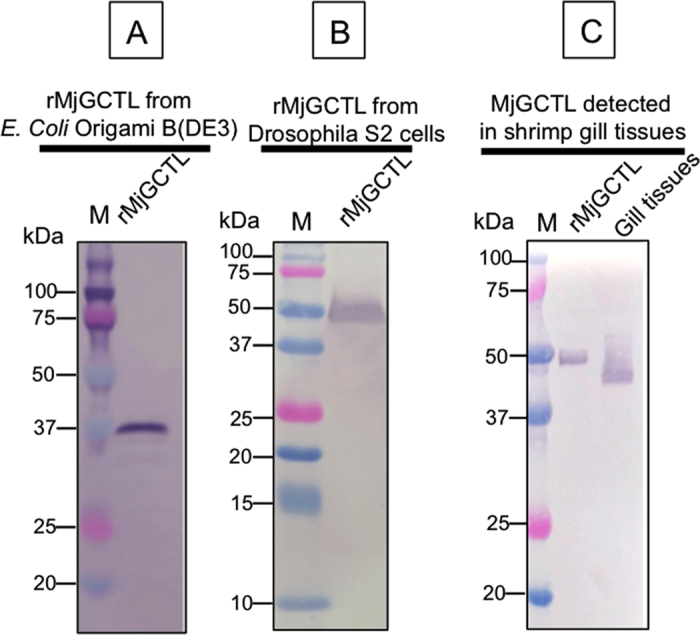
Western Blot analysis of MjGCTL (**A**) in IPTG induced *E. coli* cells detected using anti-His Lane 1: protein marker (M), Lane 2: rMjGCTL showing the detected protein around 37 kDa. (**B**) Purified rMjGCTL from *Drosophila* Schneider 2 cells. Lane 1: protein marker (M), Lane 2: rMjGCTL showing the detected protein around 50 kDa. (**C**) Detection of MjGCTL using anti-MjGCTL in rMjGCTL (lane 2) and in gill tissues (lane 3) showing the detected protein around 50 kDa and slightly below 50 kDa (40–45 kDa), respectively.

The IPTG induced *E. coli* cells yielded a rMjGCTL with a size of approximately 37 kDa (Fig. 4A). While successfully purified eluted rMjGCTL from *Drosophila* S2 cells detected by anti-V5 was as approximately 50 kDa (Fig. 4B), which is confirmed by MjGCTL detection in gill tissue that was higher than 37 kDa and slightly lower than 50 kDa (40–45 kDa) (Fig. 4C).

### MjGCTL promotes bacterial agglutination

**Figure 5:**
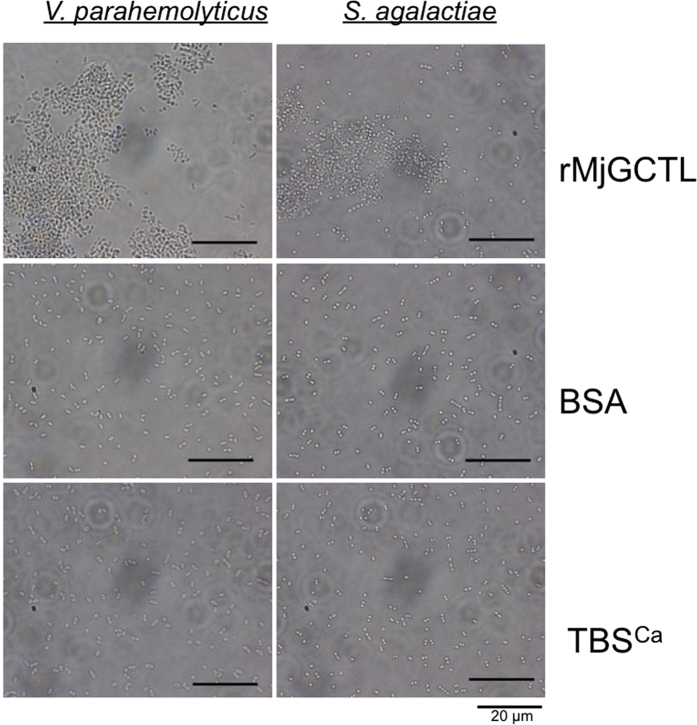
Bacterial agglutination activity of rMjGCTL with Gram-negative *V. parahaemolyticus* and Gram-positive *S. agalactiae*. Bacterial suspensions were incubated with rMjGCTL, BSA as the protein control, and TBS-Ca^2+^ (TBSCa) as the negative control.

rMjGCTL expressed by S2 cells agglutinated both Gram-negative bacteria (EGFP-*E. coli* and *Vibrio parahaemolyticus*) and a Gram-positive bacterium (*Streptococcus agalactiae*) (Fig. 5). The bacterial agglutination was concentration-dependent with a minimum agglutination concentration of 12.5 μg/mL.

### Calcium-dependent binding and carbohydrate specificity of MjGCTL

**Figure 6:**
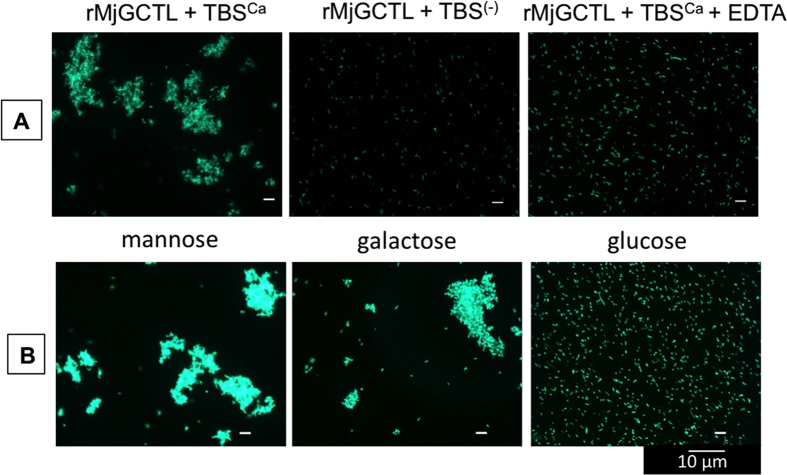
MjGCTL functions as a classical C-type lectin. (**A**) Ca^2+^-dependent agglutination activity of rMjGCTL (50 μg/ml) from *Drosophila* S2 cells with EGFP-expressing *E. coli* as influenced by the presence (TBSCa) and absence (TBS(−)) of Ca^2+^ and the addition of a chelating agent EDTA. (**B**) Agglutination inhibition by monosaccharaides Man, Gal, and Glc with *Vibrio parahaemolyticus*

rMjGCTL’s ability to agglutinate fluorescent (EGFP-expressing) *E. coli* was inhibited by the removal of CaCl_2_ or by the addition of EDTA (Fig. 6A), confirming the Ca^2+^-dependency of rMjGCTL’s agglutinating activity. Agglutination inhibition assays using different carbohydrates revealed that Man and Gal did not inhibit rMjGCTL’s ability to agglutinate *V. parahaemolyticus* but only Glucose (Glc) (Fig. 6B). Agglutination of was also inhibited by other mono- and disaccharides, and by LPS and PGN in varying concentrations [Table t1](Table 2).

### rMjGCTL’s opsonic effect enhance hemocyte encapsulation

**Figure 7:**
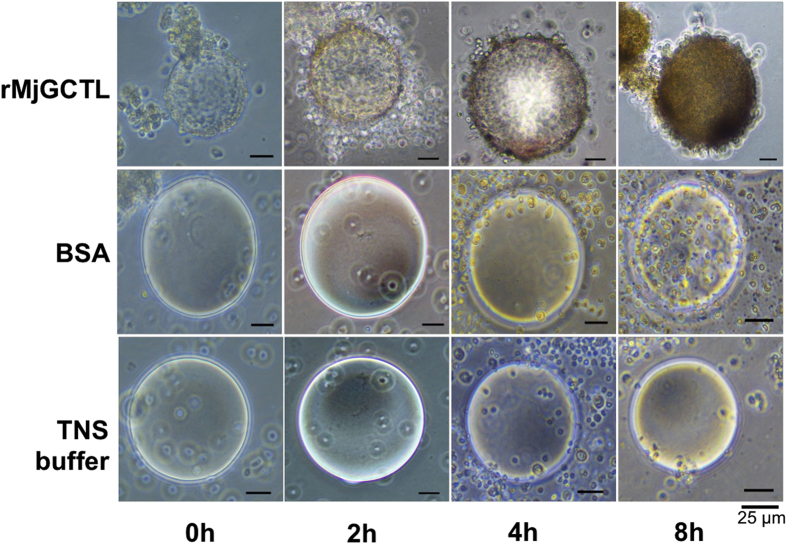
MjGCTL facilitates *M. japonicus* hemocytes encapsulation of Ni-NTA agarose beads. Incubated with rMjGCTL (50 μg/ml) from *Drosophila* S2 cells, BSA (protein control), and TNS buffer (negative control) demonstrate encapsulation by hemocyte after 0, 2, 4 and 8 hours post-incubation (hpi).

**Figure 8:**
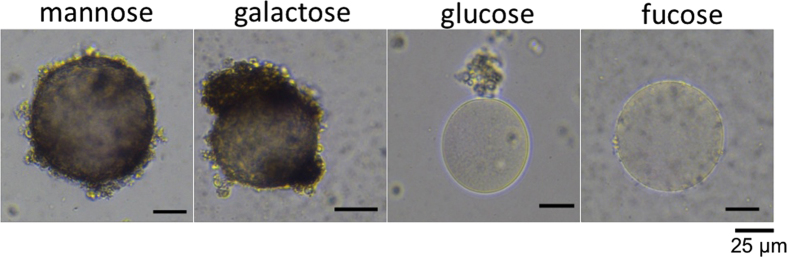
Promotion of hemocyte encapsulation is attributed to MjGCTL’s carbohydrate-binding activity. MjGCTL’s binding to hemocytes and the activation of encapsulation are inhibited by MjGCTL’s carbohydrate substrates, which is demonstrated by incubating Man, Gal, Glc, and Fuc at 100 mM with agarose beads bound to rMjGCTL (100 μg/ml) from *Drosophila* S2 cells after 8 hpi.

Hemocytes encapsulated rMjGCTL-Agarose beads immediately after they were added (0 hours post incubation (hpi)) and the binding increased from 2, 4, to 8 hpi (Fig. 7). On the other hand, no binding was observed with BSA-coated beads (protein control) or uncoated beads in TNS buffer (negative control). Melanization (indicated by the brown coloration on beads) also increased with incubation time for rMjGCTL-coated beads but not in the protein and negative controls. Results of hemocyte encapsulation inhibition by carbohydrates (Fig. 8) showed encapsulation by hemocytes were inhibited by Glc and fucose (Fuc), and not by Man and Gal. These results also show that encapsulation by hemocytes results from the binding of rMjGCTL carbohydrate recognition domain to hemocytes as demonstrated by the inhibition through competitive binding of the carbohydrate substrates Glc and Fuc.

## Discussion

Diversification of shrimp CTLDcps is manifested in their tissue distribution, domain architecture, and sugar specificity^20^. In contrast to most invertebrate lectins that are mostly expressed in the hepatopancreas, the localization of MjGCTL is in gills and stomach (Fig. 3A) which may suggest MjGCTL reside in the main entry sites and target organs of major shrimp pathogens^28^,^29^,^30^,^31^. MjGCTL functions as a classical CTL binding to carbohydrates and bacterial components (Table 2), allowing it to cause agglutination (Fig. 5) in a Ca^2+^-dependent manner (Fig. 6A). MjGCTL may also act as an opsonin, as clearly shown by the encapsulation assay (Fig. 7) and its inhibition by Glc and Fuc (Fig. 8) indicate that rMjGCTL CRD can bind to hemocytes surface carbohydrates facilitating encapsulation. MjGCTL’s being a PRR also give us an insight on the poorly understood mechanism of invertebrate hemocyte encapsulation, where MjGCTL was observed to activate the adhesive state of and recruit hemocytes to encapsulate agarose beads by binding to hemocytes (Fig. 8).

Conventionally, CTLs are classified by their CRDs into the mannose-type EPN motif or Gal-type QPD motif. In the phylogenetic analysis of the shrimp CRDs (Fig. 2), MjGCTL clustered closely with four other CTLDs containing a QAP binding motif: LvCTLD^25^, MjLdlrLec 1 and 2^27^, and MnCTLDcp 1 and 2^26^. Among these, MjGCTL shares highest identity (77%) with LvCTLD (Supporting information, Fig. 1). LvCTLD has an affinity for Gal, suggesting that QAP is a variant of QPD motif^25^. Contrary to the report on LvCTLD, our results suggest that the QAP is a new motif with a different carbohydrate-binding specificity from EPN and QPD motif. The position of MjGCTL’s CRD in the tree (Fig. 2) infers it is not associated with neither EPN and QPD nor their variants (QPT, EPS, etc.). This is corroborated by the binding specificity of MjGCTL, where the finding that MjGCTL does not bind either Man or Gal (Table 2) suggests that its specificity is completely different from that of CTLs with EPN and QPD motifs. Comparing the specificity of MjGCTL to the X-ray crystallographic analyses suggest that the binding specificity of the QAP motif is more similar to that of EPN than to that of QPD, where the QPD motif has an affinity for an equatorial/axial 3-OH/4-OH configuration of carbohydrate residues (Gal and N-acetylgalactosamine)^32^,^33^, while the EPN motif has an affinity for an equatorial/equatorial 3-OH/4-OH configuration of carbohydrate residues (Man, Glc, N-Acetylglucosamine, and fucose)^33^,^34^. Interestingly, all reported CTLDcps with QAP binding motifs are only found in shrimps, including MjGCTL, and they all contain a low-density lipoprotein receptor class A (LDLa) domain. Shrimp CTLDcps with an LDLa domain interact with viral particles^25^,^26^ or respond to viral infection such as WSSV^26^. However, in the case of MjGCTL, mRNA levels remained unchanged after WSSV infection (Fig. 3B), possibly because the LDLa domain does not interact with WSSV particles. Moreover, shrimp CTLDs not containing LDLa were found to bind with viral particles or change their mRNA transcript levels in response to viral infections^15^,^35^,^36^,^37^,^38^,^39^, thus suggesting that the LDLa domain of MjGCTL does not necessarily imply that the molecule binds or responds to viral particles, which is a characteristic of CTLD molecules. Thus, it is possible that in the case of CTLs LDLs serve as Ca^2+^-binding site, as they have been known to sequester and regulate Ca^2+^ levels^29^,^40^. However, the unchanged mRNA levels also do not mean MjGCTL does not respond to invading pathogens. This is shown by the finding that knockdown of MjHeCL, a CTL that is constitutively expressed in hemocytes, caused the bacteria to proliferate in hemolymph^41^. It is possible that just like MjHeCL, MjGCTL is constantly expressed in high levels as readily available defense molecules of the shrimp immune system.

Characterization of the domain architecture of MjGCTL demonstrates its uniqueness and binding-specificity is attributed not only to its binding motif but more importantly to its proper folding and glycosylation^6^. Here we speculate that the shift in the molecular weight detected of the purified rMjGCTL from its predicted size based on its amino acid sequence (35 kDa), may be due to the addition of carbohydrate moieties that is most likely to the presence of glycosylation. Glycosylation of MjGCTL by *Drosophila* S2 cells was expected as the sequence contains five potential N-glycosylation sites and two potential O-glycosylation sites (Fig. 1). Interestingly, the attempted production of rMjGCTL by *E. coli* expression system yielded a rMjGCTL with a protein size of about 37 kDa (Fig. 4A). This may be possible, as the *E. coli* expression system is known to have the disadvantage of producing misfolded proteins and cannot carry out post-translational modifications such as N- or O- glycosylations^42^. On the other hand, the eukaryotic expression system of *Drosophila* S2 cells are have been demonstrated to be a reliable system for proteins requiring N-glycosylations for their proper production as compared to *E. coli*^43^. This is confirmed by the detection of MjGCTL in gill tissues that revealed its actual size slightly lower than rMjGCTL from *Drosophila* S2 cells (Fig. 4C) as the latter is tagged with both V5- and His- epitope which is predicted to have a size of 3.4 kDa.

Despite the high identity to MjGCTL, LvCTLD does not function as a CTL. CTLDs are technically not lectins, and like LvCTLD they are Ca^2+^-independent. This is supported by the 3D models of MjGCTL and LvCTLD (Supporting information, Fig. 1) that show, unlike LvCTLD, the predicted locations of the Ca^2+^-binding site are co-located with QAP motif at the 5th-sheet. This is in accord with the structures of classical CTLs, in which the Ca^2+^-binding site is coupled with the sugar-binding motif in the CRD^23^,^24^. This comparison of MjGCTL and LvCTLD therefore show clearly the demarcation of CTLs and CTLDs, and thus provide a good reference for characterization of the growing number of the diverse superfamily of CTLDcps.

In summary, the characterization of MjGCTL provides further evidence of the complexity and diversity of invertebrate CTLDs. MjGCTL is a classical C-type lectin that binds to carbohydrates in a Ca^2+^-dependent manner. It functions as a PRR, recognizing non-self, through bacterial agglutination and it acts as an opsonin by binding directly to hemocytes to activate their adhesive state for their encapsulation. The sugar specificity of the QAP binding motif of MjGCTL is different from that of EPN and QPD motifs, suggesting QAP as a new binding motif. The finding that MjGCTL is almost identical to LvCTLD, yet possesses a different function, clearly demonstrates CTLDcps superfamily can be more accurately classified by their function rather than solely by their binding motif they possess. The divergent binding-motif and specificity of MjGCTL provides an insight on how invertebrate immune system possesses unique and highly specific pathogen recognition receptors through the CTLDcps.

## Methods

### Experimental Shrimp

*Marsupenaeus japonicus* shrimp weighing 10 grams were obtained from a farm in Miyazaki prefecture Japan and were acclimatized for at least 3 days before the experiment. Shrimp were kept in tanks with recirculating water maintained at 25 °C μg/mL and 35 ppt salinity.

### RNA isolation and cDNA synthesis

Total RNA was isolated from various tissues (gills, hepatopancreas, lymphoid organ, hemocytes, muscle, stomach, heart, intestine, nerve, and eye) of *M. japonicus* using RNAiso (TAKARA, Japan) that was used as template (1 μl) for cDNA synthesis using High capacity cDNA reverse transcription kit (Applied Biosystems, USA).

### Detection and sequencing of full cDNA sequence of MjGCTL

MjGCTL primers (Table 1) were designed using EST data from *M. japonicus* cDNA library. Full-length MjGCTL was amplified, cloned to pGEM T-easy vector (Promega, USA) and cloned into competent *Escherichia coli* JM109 cells and sequenced in both directions using M13 primers (Table 1). The sequences were aligned and assembled using GENETYX Ver.11.04 (GENETYX, Japan).

### Analysis of MjGCTL expression profile

MjGCTL transcripts were detected through semi-quantitative RT-PCR using 1 μl of cDNA from various tissues of *M. japonicus* using MjGCTL primers and elongation factor 1*α* (EF-1*α*) as an internal control (Table 1). Thermal cycling conditions were pre-denaturation at 95 °C for 5 min, 28 cycles of 95 °C for 30 sec, 55 °C for 30 sec and 72 °C for 1 min, and followed by final extension at 72 °C for 5 min. The PCR products were then viewed by 1% agarose gel electrophoresis.

### Cell culture of *Drosophila* Schneider 2 (S2) cells

*Drosophila* Schneider 2 (S2) cells were cultured in Schnieder *Drosophila* medium (SDM) (Invitrogen) supplemented with 10% fetal bovine serum (FBS) and antibiotics in a culture flask maintained without CO_2_ at 28 °C. S2 cells were passaged several times until reaching the optimum cell growth rate following the manufacturer’s protocol.

### Production of recombinant MjGCTL (rMjGCTL)

The cDNA sequence of MjGCTL was amplified using rMjGCTL primers (Table 1), where EcoRI and NotI sites were added to the forward and reverse primers (5′ and 3′ end), respectively. The rMjGCTL PCR was cloned then cloned into the restriction sites of expression vector pMT:BiP:V5-His C (Invitrogen, Carlsbad, CA, USA). Recombinant plasmid was then transfected using Effectene Transfection Reagent (Qiagen, Germany) following manufacturer’s instructions. Stable cell lines were selected by passaging the cells several times on SDM with 125 mg/ml of blasticidin. Following the large-scale production of the stable cell-lines, protein expression was then induced by CuSO_4_ (600 μM). One day after induction, the cells were centrifuged and rMjGCTL was purified from the supernatant using Ni-NTA agarose (Qiagen) purification column with 500 mM imidazole. The purified protein was quantified using DC protein assay (BIO-RAD) following the manufacturer’s protocol.

Prior to using S2 cells, production of rMjGCTL through bacterial cell system was also attempted through cloning MjGCTL to pCold II DNA (Takara bio, Japan) and transforming it to *Escherichia coli* (Origami B(DE3)), however the purification of the recombinant protein from large-scale production was unsuccessful.

### SDS-PAGE and Western blot analysis

Eluted proteins from *Drosophila* S2 cells were subjected to 15% SDS-polyacrylamide gel electrophoresis and transferred to a nitrocellulose membrane. The proteins were then detected with mouse anti-V5 monoclonal antibody (Thermo Fisher Scientific, USA) as primary antibody diluted to 1:5000 in 5% BSA and 0.05% Tween 20 in TBS (TBST) (blocking solution), and with anti-mouse IgG (H + L), AP conjugate (Promega, USA) diluted to 1:10000 in blocking solution. Bands were then visualized with substrate solution containing 5-bromo-4-chloro-3-indolyphosphate and nitroblue tetrazolium (BCIP/NBT, Sigma) for 5 min and washed with distilled water. On the other hand, rMjGCTL from IPTG induced *E. coli* cells were detected through 12% SDS-PAGE and Western blot analysis using monoclonal anti-His mouse IgG, however rMjGCTL were not detected upon purification.

To determine the actual size of MjGCTL in shrimp, total protein were extracted from gill tissues and was subjected to 12% SDS-PAGE and Western Blot analysis, as described above, using rabbit polyclonal anti-MjGCTL diluted to 1:5000 with blocking solution (2.5% skimmed milk in TBST) as the primary antibody and anti-Rabbit IgG(Fc), AP Conjugate (1:5000) (Promega, USA) as the second antibody.

### Bacterial agglutination assay

Agglutination was assayed by a modification of the method of Luo *et al*.^44^. The bacteria used in the assay were two Gram-negative *Vibrio parahaemolyticus* and EGFP-expressing *Escherichia coli* and Gram-positive *Streptococcus agalactiae*. Bacteria were resuspended in TBS-Ca^2+^ (Tris-HCl, pH 7, 100 mM NaCl, and 10 mM CaCl_2_) at 1 × 109 cells/ml. Ten microliters of bacteria suspension was then incubated with same amount rMjGCTL (50 μg/ml) at room temperature (25 °C) for 1 h. Agglutination were then viewed under light microscope (Nikon) and fluorescence microscopy for GFP-expressing *E. coli*. The minimum concentration of rMjGCTL that would agglutinate was determined with serial dilutions of rMjGCTL in TBS-Ca^2+^ (10 mM) and was used in agglutination assay as mentioned above. To test the calcium-dependency of rMjGCTL, agglutination was assayed using only TBS (without CaCl_2_) and also by adding EDTA with final concentration of 10 mM into TBS.

### Agglutination inhibition assay of carbohydrates

The sugar specificity of rMjGCTL was expressed as the minimum concentration of a sugar needed to inhibit agglutination. rMjGCTL was incubated with different concentrations of several carbohydrates for 1 h at room temperature prior to the agglutination assay described above. All assays were done in triplicates.

### *in vitro* encapsulation assay

The assay was done as described by Ma *et al*.^45^. BSA-coated beads and uncoated beads in TNS buffer (10 mM Tris-HCl, 140 mM NaCl, 20 mM CaCl_2_, pH 7.9) were used as positive and negative controls, respectively. Prior to adding the hemocytes, the beads were washed three times with 1 ml TNS buffer, incubated with 1 ml of His-tagged rMjGCTL or BSA (300 μM) in TNS overnight at 4 °C with gentle rotation, washed five times with 1 ml TNS and resuspended in 1 ml TNS as a 50% slurry. Hemolymph was collected from *M. japonicus* with a 23-gauge needle and syringe with 1.5 ml PBS containing 10 mM EDTA. The hemocytes were then collected, washed three times, and resuspended in 1 ml 2x Leibovitz’s L-15 medium (Invitrogen, USA). Encapsulation was assayed by adding 10 μl of the hemocyte suspension to the wells of a 24-well cell culture plate coated with 500 μl 1% agarose. One (1) μl of the beads slurry (incubated with rMjGCTL or BSA) was added to each well. Beads suspended in TNS buffer were used as a negative control. Encapsulation by hemocytes was then observed at 0, 2, 4 and 8 h post incubation (hpi) under a light microscope (Nikon).

### Hemocyte encapsulation inhibition by carbohydrates

Prior to the encapsulation assay, 100 mM of Man, Gal, Glc, and Fuc were incubated with the bound rMjGCTL and was viewed at 8 hpi. Experimentally added carbohydrates with affinity to MjGCTL will compete with that of the carbohydrate substrates of hemocytes therefore disrupting the encapsulation of the agarose beads. All experiments were done in triplicates.

## Additional Information

**How to cite this article:** Alenton, R. R. R. *et al*. Pathogen recognition of a novel C-type lectin from *Marsupenaeus japonicus* reveals the divergent sugar-binding specificity of QAP motif. *Sci. Rep.* **7**, 45818; doi: 10.1038/srep45818 (2017).

**Publisher's note:** Springer Nature remains neutral with regard to jurisdictional claims in published maps and institutional affiliations.

## Supplementary Material

Supplementary Information

## Figures and Tables

**Table 1 t1:** Primer sequences used in this study.

Name	sequence 5′-3′
MjGCTL F	GGCATGGAATAACAGCGAGT
MjGCTL R	CATTTTGGATGCTGAGCAGA
MjGCTL qRT F	GCGCACTAACCTAAACAACACTCTC
MjGCTL qRT R	CATAGAAAACGGAGAGGCACTG
M13 F	GTAAAACGACGGCCAG
M13 R	CAGGAAACAGCTATGA
EF-1*α* F	ATGGTTGTCAACTTTGCCCC
EF-1*α* R	TTGACCTCCTTGATCACACC
EF-1*α* qRT F	ATTGCCACACCGCTCACA
EF-1*α* qRT R	TCGATCTTGGTCAGCAGTTCA
EcoRIMjGCTL	GAATTCGGAATGCACGGGCGACGAAGTGGC
NotIMjGCTL	GCGGCCGCGACAGAGGTGACACAAA

**Table 2:** Carbohydrate specificity of MjGCTL.

Saccharides	Minimum inhibitory concentration
D-galactose	NI*
D-mannose	NI*
D-glucose	100 mM
Xylose	62.5 mM
Fucose	250 mM
N-Acetyl-D-galactosamine	NI*
N-Acetyl-D-glucosamine	250 mM
Lactose	250 mM
Maltose	125 mM
Sucrose	NI*
Lipopolysaccharide	62.5 μg/mL
Peptidoglycan	6.5 μg/mL

The following are the carbohydrate-binding specificity of MjGCTL displayed by the minimum inhibitory agglutination concentration of carbohydrates and bacterial components.

^*^Not inhibited (NI) at 500 mM.

## References

[b1] Maningas M. B. B., Kondo H. & Hirono I. Molecular mechanisms of the shrimp clotting system. Fish & shellfish immunology 34, 968–972 (2013). 23044383 10.1016/j.fsi.2012.09.018

[b2] Amparyup P., Sutthangkul J., Charoensapsri W. & Tassanakajon A. Pattern recognition protein binds to lipopolysaccharide and *β*-1, 3-glucan and activates shrimp prophenoloxidase system. Journal of Biological Chemistry 287, 10060–10069 (2012). 22235126 10.1074/jbc.M111.294744 PMC3322982

[b3] Hanington P. C. . Role for a somatically diversified lectin in resistance of an invertebrate to parasite infection. Proceedings of the National Academy of Sciences 107, 21087–21092 (2010). 10.1073/pnas.1011242107 PMC3000291 21084634

[b4] Loker E. S., Adema C. M., Zhang S.-M. & Kepler T. B. Invertebrate immune systems–not homogeneous, not simple, not well understood. Immunological reviews 198, 10–24 (2004). 15199951 10.1111/j.0105-2896.2004.0117.x PMC5426807

[b5] Vasta G. R., Ahmed H. & Odom E. W. Structural and functional diversity of lectin repertoires in invertebrates, protochordates and ectothermic vertebrates. Current opinion in structural biology 14, 617–630 (2004). 15465324 10.1016/j.sbi.2004.09.008

[b6] Pees B., Yang W., Zárate-Potes A., Schulenburg H. & Dierking K. High innate immune specificity through diversified c-type lectin-like domain proteins in invertebrates. Journal of innate immunity 8, 129–142 (2015). 26580547 10.1159/000441475 PMC6738811

[b7] Little T. J., O’Connor B., Colegrave N., Watt K. & Read A. F. Maternal transfer of strain-specific immunity in an invertebrate. Current Biology 13, 489–492 (2003). 12646131 10.1016/s0960-9822(03)00163-5

[b8] Roth O., Sadd B. M., Schmid-Hempel P. & Kurtz J. Strain-specific priming of resistance in the red flour beetle, tribolium castaneum. Proceedings of the Royal Society of London B: Biological Sciences 276, 145–151 (2009). 10.1098/rspb.2008.1157 PMC2614262 18796392

[b9] Sadd B. M. & Schmid-Hempel P. Insect immunity shows specificity in protection upon secondary pathogen exposure. Current Biology 16, 1206–1210 (2006). 16782011 10.1016/j.cub.2006.04.047

[b10] Cambi A., Koopman M. & Figdor C. G. How c-type lectins detect pathogens. Cellular microbiology 7, 481–488 (2005). 15760448 10.1111/j.1462-5822.2005.00506.x

[b11] Wang X.-W. & Wang J.-X. Pattern recognition receptors acting in innate immune system of shrimp against pathogen infections. Fish & shellfish immunology 34, 981–989 (2013). 22960101 10.1016/j.fsi.2012.08.008

[b12] Tassanakajon A., Somboonwiwat K., Supungul P. & Tang S. Discovery of immune molecules and their crucial functions in shrimp immunity. Fish & shellfish immunology 34, 954–967 (2013). 23059654 10.1016/j.fsi.2012.09.021

[b13] Rabinovich G. A., van Kooyk Y. & Cobb B. A. Glycobiology of immune responses. Annals of the New York Academy of Sciences 1253, 1–15 (2012). 22524422 10.1111/j.1749-6632.2012.06492.x PMC3884643

[b14] Sun Y.-D. . A hepatopancreas-specific c-type lectin from the chinese shrimp fenneropenaeus chinensis exhibits antimicrobial activity. Molecular immunology 45, 348–361 (2008). 17675157 10.1016/j.molimm.2007.06.355

[b15] Wang X.-W., Zhang X.-W., Xu W.-T., Zhao X.-F. & Wang J.-X. A novel c-type lectin (fclec4) facilitates the clearance of vibrio anguillarum *in vivo* in chinese white shrimp. Developmental & Comparative Immunology 33, 1039–1047 (2009). 19447130 10.1016/j.dci.2009.05.004

[b16] Watanabe A. . Characterization of a novel c-type lectin, bombyx mori multibinding protein, from the b. mori hemolymph: mechanism of wide-range microorganism recognition and role in immunity. The Journal of Immunology 177, 4594–4604 (2006). 16982897 10.4049/jimmunol.177.7.4594

[b17] Yu X.-Q. & Kanost M. R. Immulectin-2, a lipopolysaccharide-specific lectin from an insect, manduca sexta, is induced in response to gram-negative bacteria. Journal of Biological Chemistry 275, 37373–37381 (2000). 10954704 10.1074/jbc.M003021200

[b18] Wang X., Zhao X. & Wang J. C-type lectin binds to beta integrin to promote hemocytic phagocy-tosis in shrimp. Fish and Shellfish Immunology 6, 1684 (2013).

[b19] Schnitger A. K., Yassine H., Kafatos F. C. & Osta M. A. Two c-type lectins cooperate to defend anopheles gambiae against gram-negative bacteria. Journal of Biological Chemistry 284, 17616–17624 (2009). 19380589 10.1074/jbc.M808298200 PMC2719400

[b20] Wang X.-W. & Wang J.-X. Diversity and multiple functions of lectins in shrimp immunity. Developmental & Comparative Immunology 39, 27–38 (2013). 22561073 10.1016/j.dci.2012.04.009

[b21] Kilpatrick D. C. Animal lectins: a historical introduction and overview. Biochimica et Biophysica Acta (BBA)-General Subjects 1572, 187–197 (2002). 12223269 10.1016/s0304-4165(02)00308-2

[b22] Ghazarian H., Idoni B. & Oppenheimer S. B. A glycobiology review: carbohydrates, lectins and implications in cancer therapeutics. Acta histochemica 113, 236–247 (2011). 20199800 10.1016/j.acthis.2010.02.004 PMC3027850

[b23] Zelensky A. N. & Gready J. E. Comparative analysis of structural properties of the c-type-lectin-like domain (ctld). Proteins: Structure, Function, and Bioinformatics 52, 466–477 (2003). 10.1002/prot.10626 12866057

[b24] Zelensky A. N. & Gready J. E. The c-type lectin-like domain superfamily. Febs Journal 272, 6179–6217 (2005). 16336259 10.1111/j.1742-4658.2005.05031.x

[b25] Junkunlo K. . A novel lectin domain-containing protein (lvctld) associated with response of the whiteleg shrimp penaeus (litopenaeus) vannamei to yellow head virus (yhv). Developmental & Comparative Immunology 37, 334–341 (2012). 22214841 10.1016/j.dci.2011.12.010

[b26] Xiu Y. . Isolation and characterization of two novel c-type lectins from the oriental river prawn, macrobrachium nipponense. Fish & shellfish immunology 46, 603–611 (2015). 26208755 10.1016/j.fsi.2015.07.011

[b27] Xu Y.-H. . Two novel c-type lectins with a low-density lipoprotein receptor class a domain have antiviral function in the shrimp marsupenaeus japonicus. Developmental & Comparative Immunology 42, 323–332 (2014). 24140299 10.1016/j.dci.2013.10.003

[b28] Clavero-Salas A. . Transcriptome analysis of gills from the white shrimp litopenaeus vannamei infected with white spot syndrome virus. Fish & shellfish immunology 23, 459–472 (2007). 17337210 10.1016/j.fsi.2007.01.010

[b29] Chang P.-S., Chen H.-C. & Wang Y.-C. Detection of white spot syndrome associated baculovirus in experimentally infected wild shrimp, crab and lobsters by *in situ* hybridization. Aquaculture 164, 233–242 (1998).

[b30] Wu J. . Effects of shrimp density on transmission of penaeid acute viremia in penaeus japonicus by cannibalism and the waterborne route. Diseases of aquatic organisms 47, 129–135 (2001). 11775794 10.3354/dao047129

[b31] Lotz J. M. & Soto M. A. Model of white spot syndrome virus (wssv) epidemics in litopenaeus vannamei. Diseases of aquatic organisms 50, 199–209 (2002). 12219976 10.3354/dao050199

[b32] Kolatkar A. R. & Weis W. I. Structural basis of galactose recognition by c-type animal lectins. Journal of Biological Chemistry 271, 6679–6685 (1996). 8636086

[b33] Lee R. T. . Survey of immune-related, mannose/fucose-binding c-type lectin receptors reveals widely divergent sugar-binding specificities. Glycobiology 21, 512–520 (2011). 21112966 10.1093/glycob/cwq193 PMC3055596

[b34] Ng K. K.-S., Drickamer K. & Weis W. I. Structural analysis of monosaccharide recognition by rat liver mannose-binding protein. Journal of Biological Chemistry 271, 663–674 (1996). 8557671 10.1074/jbc.271.2.663

[b35] Song K.-K. . Cloning and characterization of three novel wssv recognizing lectins from shrimp marsupenaeus japonicus. Fish & shellfish immunology 28, 596–603 (2010). 20045060 10.1016/j.fsi.2009.12.015

[b36] Luo T., Zhang X., Shao Z. & Xu X. Pmav, a novel gene involved in virus resistance of shrimp penaeus monodon1. Febs Letters 551, 53–57 (2003). 12965204 10.1016/s0014-5793(03)00891-3

[b37] Luo T., Li F., Lei K. & Xu X. Genomic organization, promoter characterization and expression profiles of an antiviral gene pmav from the shrimp penaeus monodon. Molecular immunology 44, 1516–1523 (2007). 17055581 10.1016/j.molimm.2006.09.015

[b38] Zhao Z.-Y. . A novel c-type lectin from the shrimp litopenaeus vannamei possesses anti-white spot syndrome virus activity. Journal of virology 83, 347–356 (2009). 18945787 10.1128/JVI.00707-08 PMC2612311

[b39] Costa F. . Cloning and molecular modeling of litopenaeus vannamei (penaeidae) c-type lectin homologs with mutated mannose binding domain-2. Genet Mol Res 10, 650–664 (2011). 21523655 10.4238/vol10-2gmr999

[b40] Nykjaer A. & Willnow T. E. The low-density lipoprotein receptor gene family: a cellular swiss army knife? Trends in cell biology 12, 273–280 (2002). 12074887 10.1016/s0962-8924(02)02282-1

[b41] Wang X.-W., Xu J.-D., Zhao X.-F., Vasta G. R. & Wang J.-X. A shrimp c-type lectin inhibits proliferation of the hemolymph microbiota by maintaining the expression of antimicrobial peptides. Journal of Biological Chemistry 289, 11779–11790 (2014). 24619414 10.1074/jbc.M114.552307 PMC4002086

[b42] Demain A. L. & Vaishnav P. Production of recombinant proteins by microbes and higher organisms. Biotechnology advances 27, 297–306 (2009). 19500547 10.1016/j.biotechadv.2009.01.008

[b43] Xu L. . Post-translational modifications of recombinant human lysyl oxidase-like 2 (rhloxl2) secreted from drosophila s2 cells. Journal of Biological Chemistry 288, 5357–5363 (2013). 23319596 10.1074/jbc.C112.421768 PMC3581389

[b44] Luo T., Yang H., Li F., Zhang X. & Xu X. Purification, characterization and cdna cloning of a novel lipopolysaccharide-binding lectin from the shrimp penaeus monodon. Developmental & Comparative Immunology 30, 607–617 (2006). 16364436 10.1016/j.dci.2005.10.004

[b45] Ma T. H.-T., Benzie J. A., He J.-G. & Chan S.-M. Pmlt, a c-type lectin specific to hepatopancreas is involved in the innate defense of the shrimp penaeus monodon. Journal of invertebrate pathology 99, 332–341 (2008). 18793643 10.1016/j.jip.2008.08.003

