# Supplementary material for: Pathogen recognition of a novel C-type lectin from Marsupenaeus japonicus reveals the divergent sugar-binding specificity of QAP motif
Source: Sci Rep. 2017 Apr 4;7:45818. doi: 10.1038/srep45818 (PMC5379193; doi:10.1038/srep45818)

Pathogen recognition of a novel C-type lectin from *Marsupenaeus japonicus* reveals the divergent sugar-binding specificity of QAP motif

Rod Russel R. Alenton[1], Keiichiro Koiwai[1], Kohei Miyaguchi[1], Hidehiro Kondo[1], Ikuo Hirono[1]

[1]Graduate School of Marine Science and Technology, Tokyo University of Marine Science and Technology, Minato-ku, Tokyo, 108-8477

SUPPLEMENTARY INFORMATION:

Fig. 1 Sequence alignment of MjGCTL and LvCTLD. The amino acid sequence of MjGCTL and LvCTLD (AEH05998) were also aligned using ClustalW in Genious 6.1.8 (Biomatters Ltd., New Zealand) software. The predicted amino acid sequences of MjGCTL and LvCTLD have the same length share the same QAP motif (Fig. 1B) with a sequence identity of 77%.

```
MjGCTL              1 MKAIVLLLCIGFATALECTGDEVACGSAERCVPYRYICDFDSDCSDGSDEDPYLCWAWNN  60
                      MK  VLLLC+GFA+AL+CTGDE+AC S ERCVPYRY+CD D+DC+DGSDE P LC AW N
LvCTLD_AEH05998     1 MKRFVLLLCLGFASALDCTGDEIACTSGERCVPYRYLCDSDNDCADGSDESPDLCLAWRN  60

MjGCTL             61 TECERGSAQCLTNGRAECIPIETYCHRTQPACSGSLNRRVCSIIEDKKLVPLASIKFIPD 120
                      T+CE+G AQC  NG  +CI IE YCHRTQPAC GSL+RR+CSII++K LVPL+SI+  P
LvCTLD_AEH05998    61 TQCEKGQAQCHANGDDQCISIEAYCHRTQPACDGSLDRRICSIIKNKSLVPLSSIRLPPS 120

MjGCTL            121 NEPADAYNRSVSLGAELRTNLNNTLSHPDCPDFYTRVGDQCLSVFYVGRSSWGEARAFCK 180
                      N+P  AYN+SV LG+ELR NLN+TLSHPDCP FYTRVG QCLSVFYVG SSWGEAR+FCK
LvCTLD_AEH05998   121 NDPGVAYNKSVELGSELRLNLNSTLSHPDCPRFYTRVGGQCLSVFYVGSSSWGEARSFCK 180

MjGCTL            181 HIGGDLLSIQNASHYIDLVNHLSENQITSDFWLGGRYELDDLSWMWLDGTPMPQGTPFWS 240
                      HIGGDLLSIQN +HY+DLVNHL +N+ITSDFWLGGRYE+DDLSW WLDGTPMP+GTPFWS
LvCTLD_AEH05998   181 HIGGDLLSIQNVNHYVDLVNHLVDNRITSDFWLGGRYEVDDLSWTWLDGTPMPRGTPFWS 240

MjGCTL            241 LRRYHHCDTRNVTVAGTYQVLEANNGECYHYTQAPEDPPRGFCAAITYGKHFYMSDEDCL 300
                      LRRY  C+ RNVT+ GT +V EANNGECYHYTQAPE PP+GFCAAITY KHFYMSDEDCL
LvCTLD_AEH05998   241 LRRYDSCNPRNVTLTGTSEVREANNGECYHYTQAPETPPKGFCAAITYDKHFYMSDEDCL 300

MjGCTL            301 ADMSPLCVTSV 311
                      ADMSPLCVT+V
LvCTLD_AEH05998   301 ADMSPLCVTAV 311
```

Fig. 2 Comparison of 3d model of the carbohydrate recognition domains of MjGCTL and LvCTLD. The 3D-structures of MjGCTL and LvCTLD predicted by Swiss Model are shown in Fig. 5. The z-scores 19.637 and 19.2970, respectively, indicate good matches to the template (bovine mincle). Black arrows indicate the locations of the CRDs and green spheres indicate the locations of the bound calcium atoms predicted by Swiss Model. Note that the CRD of MjGCTL is co-located with the Ca2+-binding site while the CRD of LvCTLD is not near Ca2+-binding site. The carbohydrate-binding QAP motif is located in β5-sheet.

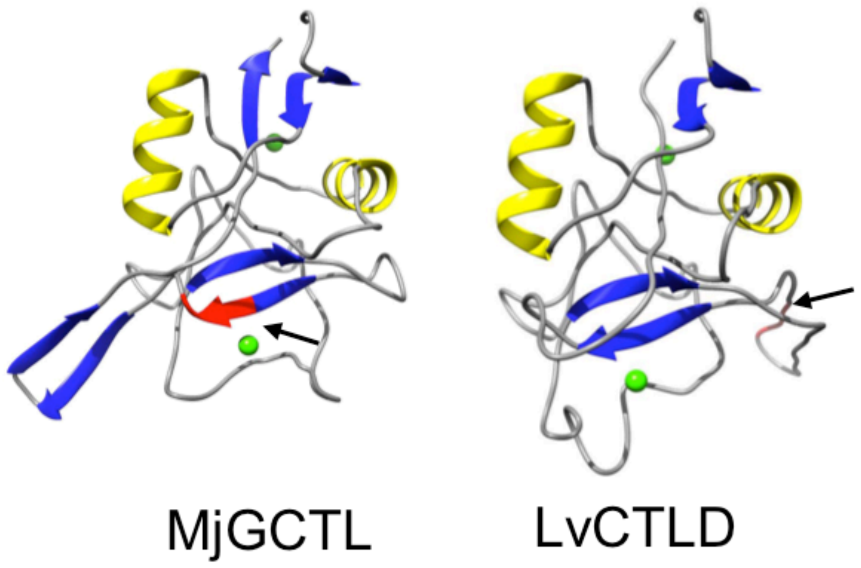

Supplement: Supplementary Information [file srep45818-s1.pdf]
